# Vagus nerve stimulation in the non-human primate: implantation methodology, characterization of nerve anatomy, target engagement and experimental applications

**DOI:** 10.1186/s42234-023-00111-8

**Published:** 2023-04-28

**Authors:** Aaron J. Suminski, Abigail Z. Rajala, Rasmus M. Birn, Ellie M. Mueller, Margaret E. Malone, Jared P. Ness, Caitlyn Filla, Kevin Brunner, Alan B. McMillan, Samuel O. Poore, Justin C. Williams, Dhanabalan Murali, Andrea Brzeczkowski, Samuel A. Hurley, Aaron M. Dingle, Weifeng Zeng, Wendell B. Lake, Kip A. Ludwig, Luis C. Populin

**Affiliations:** 1 Department of Neurological Surgery, University of Wisconsin-Madison, Madison, WI USA; 2 Wisconsin Institute for Translational Neuroengineering, University of Wisconsin-Madison, Madison, WI USA; 3 Department of Neuroscience, University of Wisconsin-Madison, 1111 Highland Ave, Madison, WI 53705 USA; 4 Department of Psychiatry, University of Wisconsin-Madison, Madison, WI USA; 5 Department of Biomedical Engineering, University of Wisconsin-Madison, Madison, WI USA; 6 Wisconsin National Primate Research Center, University of Wisconsin-Madison, Madison, WI USA; 7 Department of Radiology, University of Wisconsin-Madison, Madison, WI USA; 8 Department of Medical Physics, University of Wisconsin-Madison, Madison, WI USA; 9 Division of Plastic Surgery, University of Wisconsin-Madison, Madison, WI USA

**Keywords:** Vagus nerve stimulation, fMRI, PET, Histology

## Abstract

**Background:**

Vagus nerve stimulation (VNS) is a FDA approved therapy regularly used to treat a variety of neurological disorders that impact the central nervous system (CNS) including epilepsy and stroke. Putatively, the therapeutic efficacy of VNS results from its action on neuromodulatory centers via projections of the vagus nerve to the solitary tract nucleus. Currently, there is not an established large animal model that facilitates detailed mechanistic studies exploring how VNS impacts the function of the CNS, especially during complex behaviors requiring motor action and decision making.

**Methods:**

We describe the anatomical organization, surgical methodology to implant VNS electrodes on the left gagus nerve and characterization of target engagement/neural interface properties in a non-human primate (NHP) model of VNS that permits chronic stimulation over long periods of time. Furthermore, we describe the results of pilot experiments in a small number of NHPs to demonstrate how this preparation might be used in an animal model capable of performing complex motor and decision making tasks.

**Results:**

VNS electrode impedance remained constant over months suggesting a stable interface. VNS elicited robust activation of the vagus nerve which resulted in decreases of respiration rate and/or partial pressure of carbon dioxide in expired air, but not changes in heart rate in both awake and anesthetized NHPs.

**Conclusions:**

We anticipate that this preparation will be very useful to study the mechanisms underlying the effects of VNS for the treatment of conditions such as epilepsy and depression, for which VNS is extensively used, as well as for the study of the neurobiological basis underlying higher order functions such as learning and memory.

**Supplementary Information:**

The online version contains supplementary material available at 10.1186/s42234-023-00111-8.

## Introduction

Electroceuticals or bioelectric therapies are centered on the premise that neurological or physiological function can be influenced in a desired manner by the application of targeted electrical stimuli to the nervous system. Of the many neuromodulation therapies currently in use, vagus nerve stimulation (VNS) is one of the most successful with an estimated 100,000 patients treated (LivaNova 2016 Annual Report, 2016). First approved by the U.S. Food and Drug Administration in 1997, VNS has been shown to be a safe and effective treatment for intractable epilepsy (Ben-Menachem 2002; Landy et al. 1993; Handforth et al. 1998; Penry and Dean 1990; The Vagus Nerve Stimulation Study Group 1995) and depression (O’Reardon et al. 2006; Marangell et al. 2002; Rush et al. 2005; Sackeim et al. 2001). In addition, ongoing research (see Milby et al. 2008; Johnson and Wilson 2018 for review) has shown great promise for VNS to treat Parkinson’s Disease (Farrand et al. 2017), increase cortical plasticity (Hays et al. 2013; Engineer et al. 2011; Hulsey et al. 2016), promote functional recovery after injury to the central nervous system (Pruitt et al. 2016; Khodaparast et al., 2013; Ganzer et al., 2018), and reduce systemic inflammation (Koopman et al. 2016; Meregnani et al. 2011), but the mechanisms underlying the effects are still not understood.

Most mechanistic studies of VNS have been performed in rodents because of their ready access, and low cost. Their results, however, have been difficult to translate to humans due to vast differences in nerve anatomy (Settell et al. 2020) and central nervous system (CNS) organization (Wise 2008). To better replicate the size and anatomical organization of the vagus nerve with respect to humans, canine and swine models have been used. These large animal preparations have shown great utility in furthering our understanding of how different fiber types within the vagus are influenced by electrical stimulation (Nicolai et al. 2020; Yoo et al. 2013) and how local chemoreceptor and baroreceptor reflex circuits are modulated by the vagus (Sturdy et al. 2017; Armstrong et al. 2022; Saku et al. 2014). On the downside, these models are limited for studying the effects of VNS on the function of the CNS because they are not used for behavioral/cognitive studies. Thus, in light of the drive to understand the mechanisms underlying the effects of VNS on the CNS, there is an acute need for a large animal VNS model to study the neurobiological basis underlying higher order cognitive functions.

Here we present a new non-human primate (NHP) model for the study of the effects of VNS on the CNS. We have adapted methodology used to implant VNS electrodes in humans (Santos 2004), and describe the routing of wires for chronic experiments. Externalizing the connections to VNS electrodes is especially important given the prohibitive cost of commercially available implantable pulse generators (IPGs), and the inability of IPGs to deliver stimulation with the millisecond precision required by behavioral tasks to probe cognitive function. The effectiveness of the interface between the electrode and nerve is demonstrated in both anesthetized, and awake stimulation experiments. Under both conditions, VNS elicited robust, consistent activation of the vagus nerve, which manifested as a decrease in the rate of respiration and/or partial pressure of carbon dioxide in the expired air, but not changes in heart rate. The long-term viability of the implant is illustrated by consistent impedance readings over the course of several months. Lastly, we present data demonstrating that this implanting methodology can be used to study the effects of VNS on the function of the CNS using functional magnetic resonance imaging (fMRI) and positron emission tomography (PET).

## Methods

### Subjects

A total nine NHPs, Rhesus monkeys (*Macaca mulatta*), three female, 6–20 years old and ranging 4.8–12.5 kg were used in this study examining the anatomical organization of the cervical segment of the vagus nerve and the physiological and neural correlates of VNS. Four of the nine NHPs were used for anatomical and histological characterization of the left cervical vagus nerve. Two of the nine NHPs were used in acute, terminal experiments to adapt the methodology to implant FDA approved VNS electrodes to the Rhesus model, carry out intraoperative measures of engagement between the nerve and electrode and for anatomical and histological characterization of the left cervical vagus nerve. Finally, the remaining three NHPs were implanted with FDA approved VNS electrodes for long term, chronic experiments. NHPs used in terminal experiments or for histological characterization were scheduled for euthanasia by the Wisconsin National Primate Research Center (WNPRC) for reasons unrelated to this study. All procedures were approved by the University of Wisconsin Institutional Animal Care and Use Committee (IACUC) and the U.S. Army Medical Research and Development Command Animal Care and Use Review Office and were in accordance with the National Institutes of Health’s *Guide for the Care and Use of Laboratory Animals*.

### Surgical implantation of VNS Electrode

**Fig. 1:**
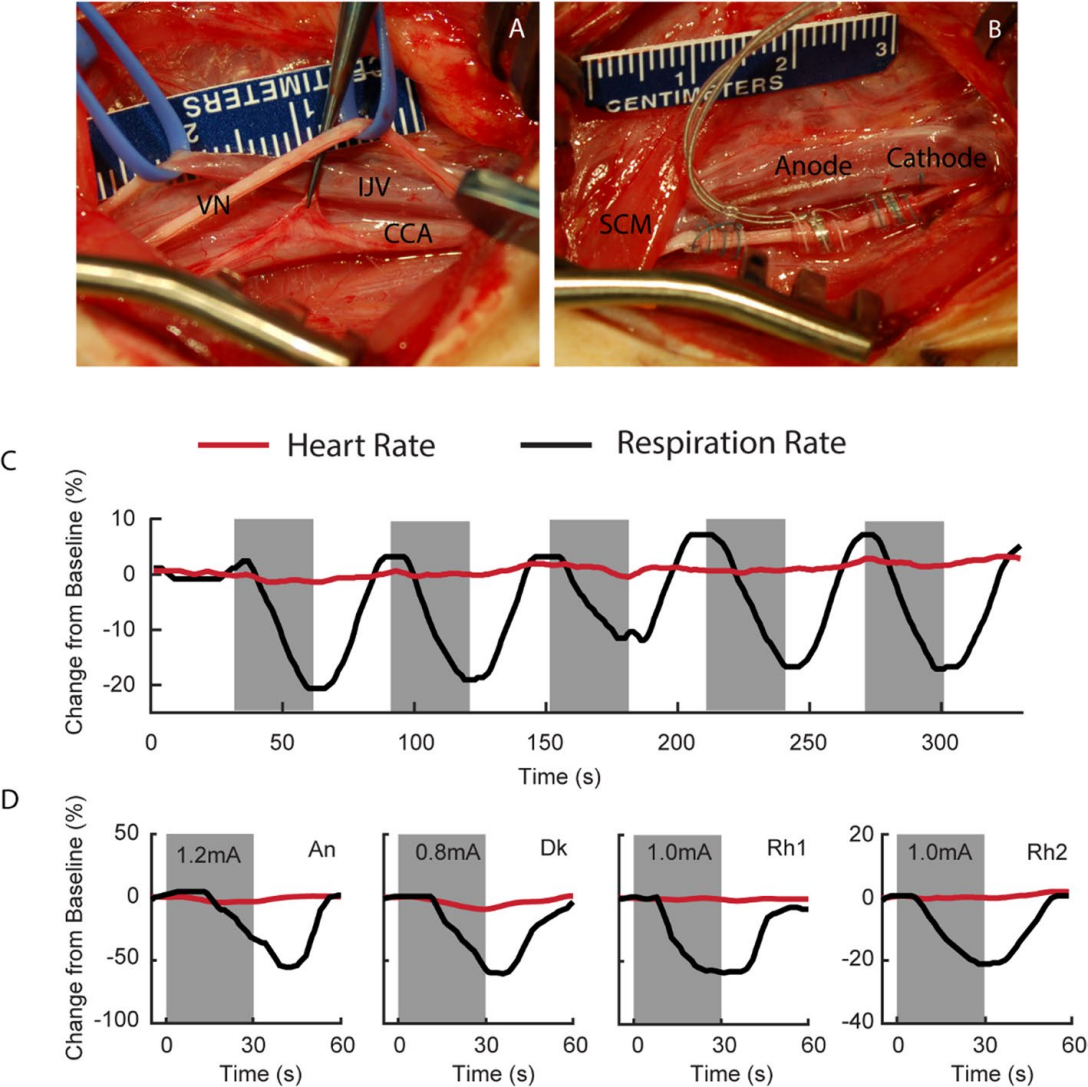
VNS electrode implantation and intraoperative verification of target engagement. **A** Surgical field following identification and careful dissection of the carotid sheath exposing the common carotid artery (CCA), internal jugular vein (IJV) and vagus nerve (VN). **B** Implantation of bipolar stimulation electrode on the vagus nerve with the cathode placed caudally to the anode. Notice the sternocleidomastoid (SCM) crossing the surgical field. **C** Representative time series of the response of heart rate (red) and respiration rate to VNS. Periods of VNS are represented by gray bars. **D** Intraoperative VNS was used to verify proper engagement between the VN and stimulating electrode. Average evoked responses for NHPs An, Dk, Rh1 and Rh2 demonstrate that 30 s of VNS (gray bars) evokes a robust, sustained parasympathetic response manifest as a greater than 20% reduction in the rate of respiration (black traces), with no apparent change in heart rate (red traces)

Five Rhesus monkeys (one female, 8–16.7 years old, weighing 8–12.5 kg) were implanted with stimulating electrodes on the left cervical vagus nerve. Two were used in acute experiments while the remaining 3 were used in chronic studies. The surgical procedures were carried out under propofol anesthesia (0.1–0.6 Mg/kg/min IV), which was chosen because it enables the detection of parasympathetic activation by stimulation of the vagus nerve in respiratory rate while in surgery. Bipolar, 2 mm inner diameter cuff electrodes (LivaNova, PerenniaFLEX Model #: 304), were implanted based on methodology adapted from Santos (2004). The electrode has two contacts, each having a geometric surface area of 6mm^2^ (6 mm x 1 mm contact dimensions) with 7 mm inter-contact spacing. Briefly, the subjects were positioned with a small towel roll placed transversely under the scapulae to achieve slight extension of the neck. The head was turned slightly to the right such that the left side of the neck was up. The incision site was marked at the level of the cricothyroid membrane on the left side of the neck (~ 3 cm rostral to the clavicle) and a 6 cm long incision was made transversely, relative to the neck, after the area was infiltrated with local anesthetic. The skin flaps were undermined subcutaneously above the platysma muscle and cautery was used to incise the platysma muscle in a rostro-caudal direction along the orientation of the fibers. Blunt dissection was used to locate the anterior edge and separate the sternocleidomastoid muscle from the sternohyoid muscle to expose and identify the carotid sheath. The carotid sheath was carefully opened exposing the internal jugular vein, vagus nerve, and common carotid artery (Fig. 1A; IJV, VN and CCA, respectively). Blunt dissection was used to isolate the vagus nerve from the vein and artery, and vessel loops were used to optimize exposure of the nerves. The sympathetic trunk was identified and excluded from the vagus nerve and the implants. The cervical sympathetic trunk was found in one of two different configurations in relation to the vagus nerve. In the most common (4/5), the cervical sympathetic trunk was small and could be completely isolated from the vagus with standard microsurgical dissection. In the less common configuration, the cervical sympathetic trunk was merged with the vagus nerve, in the middle of the distal portion. This configuration required a more extensive and delicate dissection, opening the adventitia / epineurium, to isolate each of them. Approximately 3 cm of the vagus nerve was exposed within the carotid sheath and the electrode was placed on the vagus nerve, making sure that the cervical sympathetic trunk was excluded from the cuffs. The distal electrode coil (Fig. 1B, Cathode) was placed first followed by the middle (Fig. 1B, Anode) and the anchor coil. The lead portion of the VNS electrode was then anchored in place to the omohyoid and the sternocleidomastoid muscles using 3 separate silicone anchors that wrap around the wire.

Implantable pulse generators are unable to deliver VNS the temporal precision required by modern behavioral paradigms as they do not permit closed loop stimulation. As such, the lead wire must be externalized to connect to a stand-alone stimulator. For this purpose the lead wire for the cuff electrodes was modified before the surgery by removing the connector provided by the manufacturer, exposing the wires connected to each stimulating electrode and attaching each one to separate pin of an 18 pin, nickel-free, Omnetics connector (A70242-001). The connection between the individual lead wires and connector were insulated with Kwik-Sil Silicone Elastomer (World Precision Instruments, Sarasota, FL). In the first two experiments, carried out acutely on animals slated for euthanasia by the WNPRC for reasons unrelated to this study, the lead wire was routed through incision in the neck and the incision closed around the wire. For the three subjects assigned to long-term, chronic studies, the lead wire was tunneled subcutaneously from the cervical incision to the interface of the skin and a previously prepared headcap and secured into a small chamber that was attached to the headcap using dental acrylic. The routing of the cables to the headcap is shown in Fig. 1C. In these subjects the platysma and subcutaneous layer were first closed using interrupted sutures of 3–0 Vicryl. Finally, the skin was closed using subcuticular sutures of 3–0 Vicryl in order to leave no suture material exposed that could be disturbed by the monkey. This method permits the study of the effects of VNS on a variety of physiological processes in awake and behaving NHPs.

### Verification of engagement between nerve and electrode

#### Intraoperative measurements

Proper engagement between the implanted electrode and the vagus nerve was assessed using two methods. First, we measured the impedance of the interface between the nerve and electrode at 1 kHz using an IZ2H neural stimulator (Tucker-Davis Technologies, Alachua, FL). Second, we measured the effect of VNS on the cardiovascular and respiratory systems. Specifically, we measured the physiological response to 30 s of VNS (cathode leading, symmetric, biphasic pulses with frequency = 30 Hz, phase duration = 100 µs, amplitude = 200 – 2000µA, 0 ms interphase delay) preceded and followed by at least a 30 s period of no stimulation. Stimulation was applied up to 5 times in each subject at each amplitude. Heart rate, respiration rate, oxygen saturation and arterial blood pressure were measured continuously using a Cardell Touch Veterinary Monitor (Midmark Co, Versailles, OH) and recorded on video, then transcribed with a 1 s sampling rate.

#### Awake measurements

Vagus nerve engagement in the awake NHPs was verified in stimulation sessions that began approximately two weeks after the electrode was implanted. Subjects sat in a primate chair with their heads restrained. A LifeSense TableTop monitor (Nonin Medical Inc, Plymouth, MN) was used to measure heart rate, respiratory rate, oxygen saturation and partial pressure of carbon dioxide (PCO_2_) in the expired air; the signals were digitized at sampling rate of 4 Hz. A veterinarian lingual sensor was gently clipped to the ear of the subjects to measure oxygen saturation and heart rate, and a plastic tube placed in front of one nostril to measure respiration rate and PCO_2_. After recording 60 s of physiological parameters with no stimulation, VNS was applied for 10 s (cathode leading, symmetric, biphasic pulses with frequency = 30 Hz, phase duration = 100 µs, amplitude = 100 – 1200µA, 0 ms interphase delay) immediately followed by a 30 s recovery period. In each session, stimulation was applied up to 5 times to each subject at each amplitude. Subjects were closely monitored and stimulation was immediately halted at the first sign of side effect. Simulation current was then reduced on subsequent trials. At the end of each session, NHPs were returned to their home cage. No stimulation was provided in the home cage.

#### Data analysis

The effects of VNS on physiological parameters from both the intraoperative and awake sessions were analyzed using Matlab (Mathworks, Natick, MA). In both datasets, raw time series were smoothed using a 5-point moving average filter. The envelope of the PCO_2_ was computed by locating the peaks in the raw PCO_2_ time series and then fitting a piecewise cubic polynomial (Matlab function pchip) to resulting data points. During intraoperative sessions, stimulation epochs, aligned to the start of stimulation, were extracted from 5 s before the start of stimulation to 30 s after stimulation ended and averaged. In the awake preparation, the timing of the VNS evoked responses was much more variable than under anesthetized conditions, thus we aligned the extracted epochs to the maximum deviation of the response from baseline and averaged the responses across stimulus presentations. Finally, the percent change from the 5 s baseline period was computed for each physiological parameter. Dose response curves (DRCs) were computed. One-way ANOVAs with post-hoc t-tests were used to assess the effect of current amplitude on the peak percent change in each physiological parameter. The stimulation threshold for eliciting a change in measured physiological variables was quantified as the lowest stimulation current to elicit a significant change from baseline.

### Histological examination of vagus nerve

Segments of the left cervical vagus nerve (approximately 30 mm in length) centered at the location of electrode implantation were harvested and fixed in 10% neutral buffered formalin at 4 °C overnight and then transferred to 70% ethanol. Nerves were processed for paraffin embedding. A transverse cut was made at the mid-point of the processed nerve, then each half was embedded in paraffin with the cut surface face down. Transverse serial sections were then obtained in both proximal and distal directions. Sections were stained for hematoxylin and eosin (H&E) and Gömöri’s trichrome. Whole slides were scanned at 40 × magnification using a PathScan Enabler IV (Meyer Instruments, Huston, TX). The entirety of the nerve area including surrounding collagen was outlined and measured on trichrome stained sections using the “freehand selections tool” of the ImageJ software (Abramoff et al. 2004; Schneider et al. 2012; Rasband and ImageJ, 1997–2018) to obtain the “total section area” (TSA). The area of all nerve fascicles was measured in the same manner. The “total fascicle area” (TFA) was obtained by adding all fascicle areas. The TFA was subtracted from TSA to obtain extracellular matrix (ECM) area or “total collagen area” (TCA). All fascicles within a section were counted manually and the size of the nerve was measured along the long and short axes of the ellipse that described the shape of the VN in cross-section.

### Behavioral testing

To evaluate the viability of the preparation for the study of the effects of VNS on cognitive function we used an oculomotor based behavioral choice task, which was well established in this laboratory. NHPs were trained to proficiency prior to implantation of the VNS electrode. Here, in each trial the subject indicated his choice by making a saccadic eye movement to one of the figures; further details pertaining to the task training, and modeling are found in Rajala et al. (2015). Briefly, the task required the subject to choose between a small reward delivered soon after the selection was made, a condition referred to as SS, and a larger reward delivered later after a delay of up to 16 s, a condition referred to as LL, based on a series of images, the meaning of which the subjects learned during training. For this proof of principle study VNS was administered at three different times during the course of the task, in different sessions, at the time of each stimulus presentation, and coinciding with the delivery of each reward in the SS or LL condition. The stimulation parameters were chosen to mimic experiments investigating the used of VNS to modulate plasticity in the CNS (Engineer et al. 2011; Hays et al. 2013; Ganzer et al. 2018; Kilgard et al. 2018) and consisted of 8, 250μA cathode leading, symmetric, biphasic pulses (200 μs per phase), at 30 Hz.

### MR imaging and image analysis

**Fig. 2:**
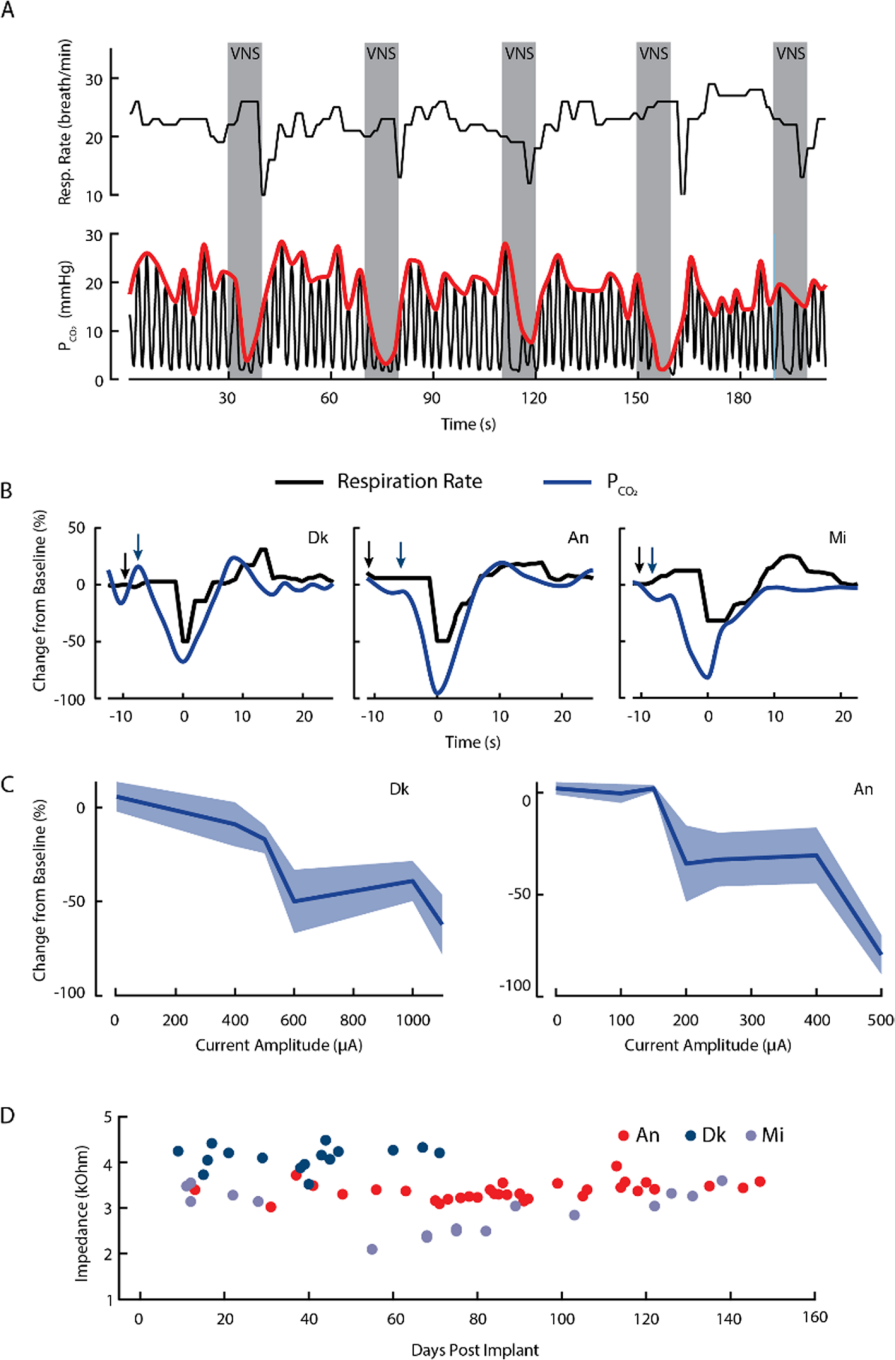
Awake verification of target engagement and electrode reliability/stability. **A** Representative measurements of heart rate, respiration rate and partial pressure of carbon dioxide (P_CO2_) during consecutive periods of VNS (10 s on, 30 s off) for NHP Dk 39 days post implant. Gray bars denote the period VNS is turned on. Similar to the anesthetized, intraoperative testing, we see noted parasympathetic effects on the rate of respiration. This depressive effect on respiration is plainly visible in the raw P_CO2_ time series (black) and its envelope (red trace). **B** Average change in respiration rate (black trace) and P_CO2_ (blue trace) for NHPs Dk, An and Mi measured 39, 86 and 122 days post implant, respectively. Stimulation amplitudes were 1000µA, 500µA and 1000µA for Dk, An and Mi, respectively. Again, we find robust parasympathetic effects on respiration but not heart rate, with the most profound effect being seen in P_CO2_. Arrows represent the average time VNS was applied to evoke these average responses. **C** Dose response curves demonstrating changes in P_CO2_ as a function of stimulation current amplitude. Greater levels of stimulation resulted in larger decreases in P_CO2_ during stimulation. **D** Impedances of the VNS electrode, measured at 1 kHz, remain stable throughout the duration of the experiment

To validate the potential of the preparation for the study of neural correlates of VNS, we performed functional MRI in one monkey during stimulation using previously described methodology (Birn et al. 2019). Briefly, the monkey sat in a custom MR compatible primate chair and was transported to the 3.0 T MRI scanner (GE Signa PET/MR, GE Heathcare, Waukesha, WI). Upon arriving at the scanner facility, the chair was slowly tilted until the monkey was in the sphinx position where its head was restrained, and a 3 inch transmit-receive MR loop (Behzadnezhad et al. 2019) coil was placed above the subject's head for imaging using a custom adaptor that attached to the head-post implanted on the NHP’s head. The animal was then inserted into the bore of the scanner tail first. Blood oxygen level dependent (BOLD) contrast was used to image the hemodynamic related changes evoked by VNS (cathode leading, symmetric, biphasic pulses with frequency = 30 Hz, phase duration = 100 µs, amplitude = 700 µA) in the awake subject (Suminski et al. 2018). A current amplitude of 700 µA was chosen to minimize the impact of changes in respiration on BOLD imaging, as the threshold for robust changes in respiration rate/PCO_2_ was 1 mA in this NHP (see Fig. 2B, Mi). VNS was applied in a blocked design paradigm having 30 s of stimulation followed by 30 s of baseline (i.e. no stimulation). A total of 10 periods of stimulation were presented during each 10-min imaging run. Physiological measures were monitored and recorded as described in the Awake Measurements section above.

The structural T1-weighted image volume was first re-oriented to correct for the nonhuman primate having been scanned in the sphinx position. Signals from non-brain tissue were then removed using AFNI’s 3dSkullStrip. To improve the skull-stripping performance, particularly in the frontal lobe, the anterior–posterior dimension was rescaled (reduced) by a factor of two prior to brain extraction. This reduced the curvature of the brain in the frontal lobe, resulting in improved brain extraction in this region. The dimensions were then adjusted back after brain extraction. The structural image was then aligned to a template/atlas brain (McLaren et al. 2008).

Functional MRI time series were first corrected for B0-field distortions using FSL’s topup, and reoriented to correct for the NHP having been scanned in the sphynx position. The first 3 image volumes were discarded to allow the magnetization to reach steady state. fMRI data were then slice-time corrected (AFNI’s 3dTshift), and motion corrected using rigid-body realignment (3dvolreg). Functional data was warped into the template space by applying a transformation computed between the native structural image and the template. Finally, the data were spatially smoothed with a Gaussian kernel with a full-width at half-maximum (FWHM) of 4 mm. Volume-to-volume motion (framewise displacement, FD) was determined by computing the Euclidean norm of the temporal difference of the estimated motion parameters, with translations in mm and rotations in degrees.

Areas of activation induced by the VNS stimulation were determined using multiple linear regression. The three runs with stimulation were temporally concatenated. The ideal task timing (a blocked design of 30 s stimulation alternated with 30 s of baseline) was convolved with a gamma-variate function (AFNI’s “BLOCK” function). The 6 realignment parameters, as well as a constant and linear trend for each of the 4 runs, were used as additional nuisance regressors. In order to evaluate the potential influence of respiration-related fMRI signal changes, the regression was repeated by including the average signal over the whole brain (global signal), the end-tidal CO2 convolved with a gamma-variate hemodynamic response function, and the temporal derivatives of these waveforms as additional nuisance regressors. Volumes with FD > 0.5 mm were censored (ignored) in the regression. The locations of significant activation were determined by warping the Calabrese parcellation to the McLaren template.

### PET imaging and analysis

To evaluate the suitability of this VNS preparation for use with PET, the radioligand [11C]raclopride, a D2/3 selective antagonist was used to measure changes in dopamine dynamics brought about by VNS. Simultaneous PET/MR imaging (GE Signa PET/MR, GE Heathcare, Waukesha, WI) with and without VNS stimulation (frequency = 30 Hz, phase duration = 100 µs, amplitude = 1 mA, 30 s on/30 s off) was acquired in one NHP anesthetized with propofol (constant rate infusion: 0.3 mg/kg/hr). The subject lay prone on the scanner bed facing out the front of the magnet bore. The PET scan was 60 min long with VNS administered after the tracer had reached steady state, at approximately minutes 22–60. Data were reconstructed to yield attenuation, scatter, and decay-corrected dynamic images with 1-min time frames. The reconstruction parameters were: TOF-OSEM, 28 subsets, 2 iterations, 256 × 256 matrix, 89 slices, 300 mm FOV, 1.17 × 1.17 × 2.8 mm^3^ pixel size, 4 mm FWHM post filtering, 60-min duration. An MR-based attenuation correction, supplemented with a fused CT image of the animal positioning and support equipment to account for attenuation and scattering of additional equipment present in the bore, was used. For anatomical localization, the structural T1-weighted MR images were first transformed into the MNI CBC15 rhesus macaque atlas space (Calabrese et al. 2015), then Paxinos atlas labels were transformed back into PET space and used to define striatum (caudate and putamen) and cerebellum. Relative standardized uptake values (SUVR) were then computed by normalizing to the cerebellum, and plotted in the form of a time-activity curve (TAC) to visualize differences in dynamic tracer binding in the presence of VNS.

## Results

The results presented here represent proof of concept that implantation of vagus nerve cuff electrodes in a rhesus macaque model show long term viability, engagement of the nerve, and, given engagement with stimulation, examples from single subjects illustrating a range of experimental techniques that can be used in conjunction with VNS in this preparation. Breathing rate and heart rate changes are used as measures demonstrating vagal engagement resulting from stimulation in both the intraoperative and awake states. The amplitudes of stimulation selected for study were based on previously published research examining the effects of VNS on the CNS (Engineer et al. 2011; Morrison et al. 2019; Loerwald et al. 2018; Borland et al. 2016; Kilgard et al. 2018). We then show an example of application for behavioral studies, in which stimulation can be administered at specific task epochs, as well as imaging studies including fMRI and PET, to characterize brain activity that may result from stimulation.

### Intraoperative characterization of nerve engagement

The integrity of the electrodes was verified before closing the implant area in all monkeys. First, the impedance between the newly implanted electrodes and the nerve was measured at 1 kHz. Across all subjects the interoperative impedance averaged 2.39 ± 1.04 kΩ (mean ± sd). VNS applied to the left cervical vagus decreased respiration rate (-49.6 ± 19.9% with respect to baseline) beginning approximately 15 s after the start of stimulation and then returning to baseline after stimulation was terminated (Fig. 1D, black traces). The threshold to elicit this effect was 1.0 ± 0.16 mA across all the subjects. We found that VNS caused only a minimal reduction in heart rate (-4.1 ± 3.9% with respect to baseline) (Fig. 1D, red traces).

### Chronic characterization of nerve engagement

Studying the effects of VNS in a behaving NHP requires a chronic preparation with an implant that remains viable for prolonged periods of time. Furthermore, to avoid repeated administration of anesthesia, we assessed the function of the electrode while the NHPs were awake. Similar to the intraoperative sessions, we measured the effects of VNS (see methods for details on stimulation parameters) on heart rate and respiration rate, but here we additionally measured PCO_2_ in the expired air (Fig. 2A).

VNS had variable effects on the different physiological measures and the shorter stimulation time (10 s) resulted in some variability in the timing of the stimulation-evoked responses (Fig. 2A; respiration rate and PCO_2_ time series). Similar to the intraoperative results, we found that VNS evoked changes in both respiration rate and PCO2, but only a modest reduction in heart rate, which did not reach significance (Fig. 2B). Specifically, we observed a decrease, with respect to baseline, in respiration rate (-49.5 ± 11.7%, -49.2 ± 13% and -33.8 ± 16.8% for Dk, An and Mi, respectively) and PCO_2_ (-68.2 ± 29.5%, -96.1 ± 6.5% and -88.5 ± 5.0% for Dk, An and Mi, respectively), but no change in heart rate (-5.1 ± 5.1% and -5.6 ± 5.3% for Dk and An, respectively). Dose response curves for Dk and An demonstrated greater decreases in PCO_2_ (Fig. 2C) as current amplitude increased. ANOVA found a significant effect of current amplitude on PCO_2_ for both Dk (F_5,24_ = 4.5, *p* = 0.0048) and An (F_6,28_ = 7.3, *p* = 9.167e^−5^). Thresholds to evoke significant reductions in PCO_2_ were 600 and 500µA for Dk and An, respectively. The response of respiration rate to stimulation was more variable than PCO_2_ with ANOVA finding a significant reduction in respiration rate caused by stimulation in An (F_6,28_ = 8.96, *p* = 1.7e^−5^) but not Dk (F_5,24_ = 0.93, *p* = 0.48). The threshold to evoke a significant reduction in An was 500µA (Fig. S1).

Lastly, the impedance of the electrode/nerve interface remained stable over the period of chronic implantation (Fig. 2D; 71, 147 and 140 days at the time of this report for Dk, An and Mi, respectively). On average, the measured impedance was 4.11 ± 0.24 kΩ and 3.37 ± 0.18 kΩ for Dk and An, respectively.

### Histology

**Fig. 3:**
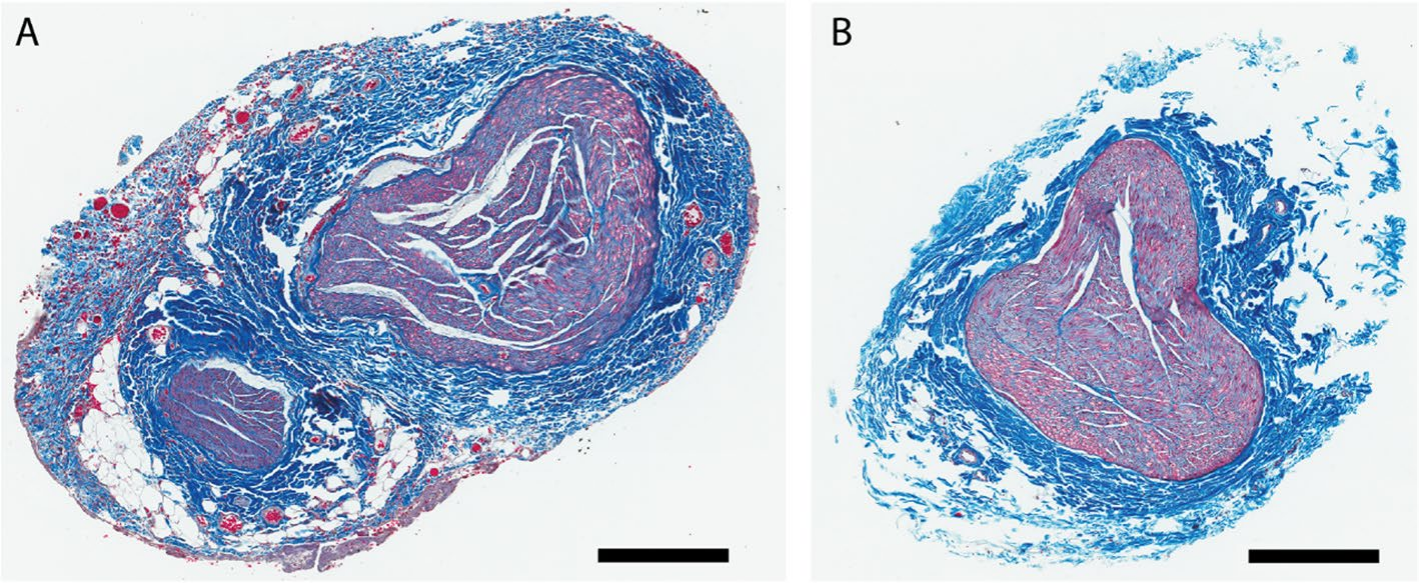
Histological examination of the NHP vagus nerve. **A** and **B** Gomori trichrome stain of cross sections of the cervical VN from two subjects showing distinct anatomical structure. Each sample contains a large fascicle with qualitatively similar morphology but one sample (panel **A**) contains a second smaller fascicle. Scale bars indicate a length of 300 µm

Histological evaluation of the left vagus nerve in NHPs was performed on portions of the nerve obtained from cervical implantation sites. Table 1 describes demographic and anatomical details for each NHP. Gömöri’s trichrome provides a clear delineation between the collagen (blue) and the myelinated axons (purple) constituting the supporting and functional components of the nerve, respectively (Fig. 3A and B). The average TSA of the cervical vagus is 1.86 ± 0.54 mm^2^. The majority of the area consisted of collagen (TCA: 1.21 ± 0.36 mm^2^, 65%), but also consisted of vasculature and adipose tissue to a far lesser degree. The majority of the collagen constituted the epineurium surrounding the smaller, functional fascicular area of the vagus nerve (TFA: 0.65 ± 0.20 mm^2^, 35%). On average the cross-section of the VN measured 1.79 ± 0.39 mm and 1.27 ± 0.14 along the long and short axes, respectively with a total of 1.67 ± 0.74 fascicles. Half the samples exhibited smaller second or third fascicles (Fig. 3A).

**Table 1:** Demographic and histological details for the NHP vagus nerves examined. Cross sectional areas of nerve features are represented as the total area as well as that of the fascicular bundles and the supporting collagen structures. Most nerves were not circular, thus the diameter is measured along the short and long axes

				**VN Area**				**VN Diameter**	
**Animal ID**	**Sex**	**Age**	**Weight (kg)**	**Total (mm^2)**	**Fascicular (mm^2)**	**Collagen (mm^2)**	**# Fascicles**	**Long**	**Short**
Rh1	F	7.5	4.83	1.124	0.34	0.784	1	1.063	1.003
Rh2	M	6.3	9.5	1.911	0.827	1.084	1	1.775	1.313
Rh3	M	6.6	11.8	2.871	0.939	1.932	3	2.347	1.46
Rh4	F	20.5	6.89	1.797	0.628	1.169	2	1.809	1.264
Rh5	M	8.3	12.48	1.461	0.504	0.957	1	1.742	1.367
Rh6	F	16.7	9.63	1.971	0.663	1.308	2	2.012	1.245

### Effects of VNS on behavioral choice and brain physiology

We evaluated the viability of the preparation for the study of the effects VNS on the function of the nervous system using an oculomotor based behavioral choice task, fMRI and PET. Data sets collected with each technique are presented as a proof of concept.

**Fig. 4:**
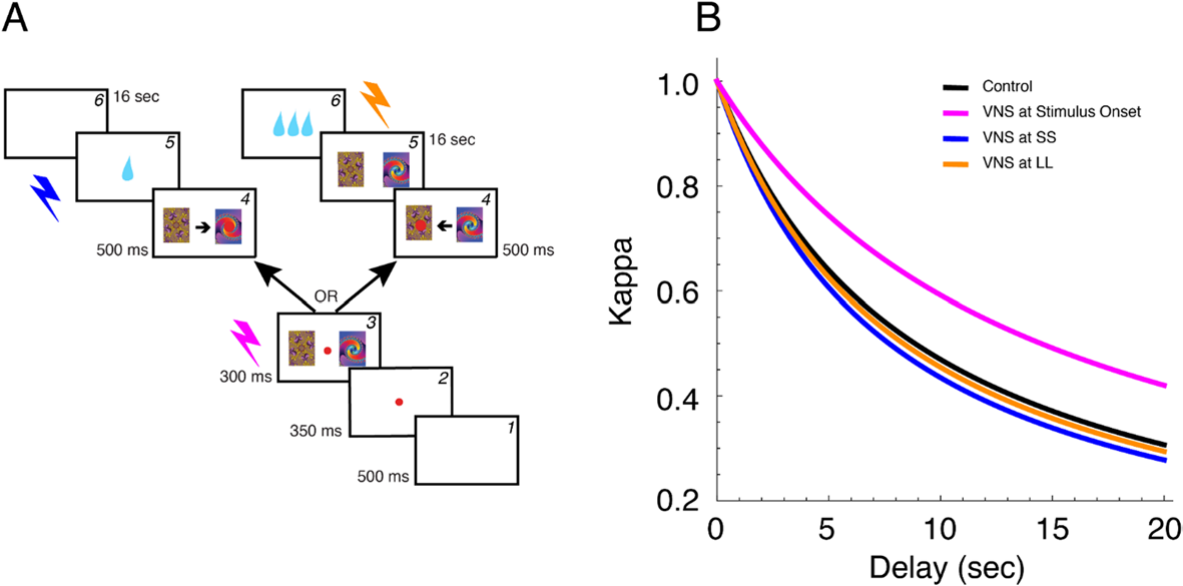
Effect of VNS on choice. **A** Schematic representation of the inter-temporal reward choice task. (*1*) The subject was required to fixate on a red dot presented in the middle of the screen, straight ahead for 350 ms, at which point (*2*) two fractal images representing the SS and LL were displayed to the left and right of the fixation point while the subject maintained fixation. (*3*) Upon offset of the fixation point, the signal to respond, the subject was expected to make a saccadic eye movement to one of the two fractal images, indicating his choice. (*4*) When the eye-tracker detected the subject’s eyes within the acceptance window of one of the images, a large red dot was overlaid on the fractal, indicating to the subject his choice. (*5*) In this example, the subject chose the SS (left image), in which case the reward was delivered immediately and a 16 s wait would precede the start of the next trial. Conversely, had the subject chosen the LL (right image), a delay of 16 s would have preceded the delivery of the reward and the onset of the next trial would have followed shortly after. Both images remained on the screen for the duration of the delay period. **B** Discounting values (Kappa) for control and three VNS conditions: VNS paired with stimulus onset, with SS and with LL. In this single session, the value of Kappa was smaller than in the other three conditions, indicating that the NHP was more patient in evaluating his choices

We demonstrate the ability to integrate VNS in a choice task by delivering a short pulse train (8 pulses at 30 Hz for a total duration of 266 ms) paired with stimulus presentation or reward. The subject’s selections in the behavioral choice task (Fig. 4A; see Behavioral Testing for task description) were fit using a hyperbolic discounting function and a probability choice model using maximum likelihood (Rajala et al. 2015) to estimate the discounting constant Κ (Kappa). The Κ values from a control session and the three experimental conditions—one session with VNS delivered at the time of stimulus presentation, one session with VNS delivered at the time of reward to reinforce the SS selection, and another session with VNS delivered at the time of reward reinforce the selection of the LL option, are plotted in Fig. 4B. Larger values of Κ indicate a preference for smaller immediate rewards compared to larger delayed rewards. In the single stimulation sessions shown in Fig. 4B, VNS reduced the value of Κ relative to control when presentation was paired with stimulus presentation, but VNS paired to the time of reward presentation, at SS or LL, seemed to have no effect. As these data were collected from a single subject as an example of behavioral applications for this technique, definitive statements on the effects of VNS on the mechanisms underlying in a choice task will be possible after replication of the study in multiple subjects.

**Fig. 5:**
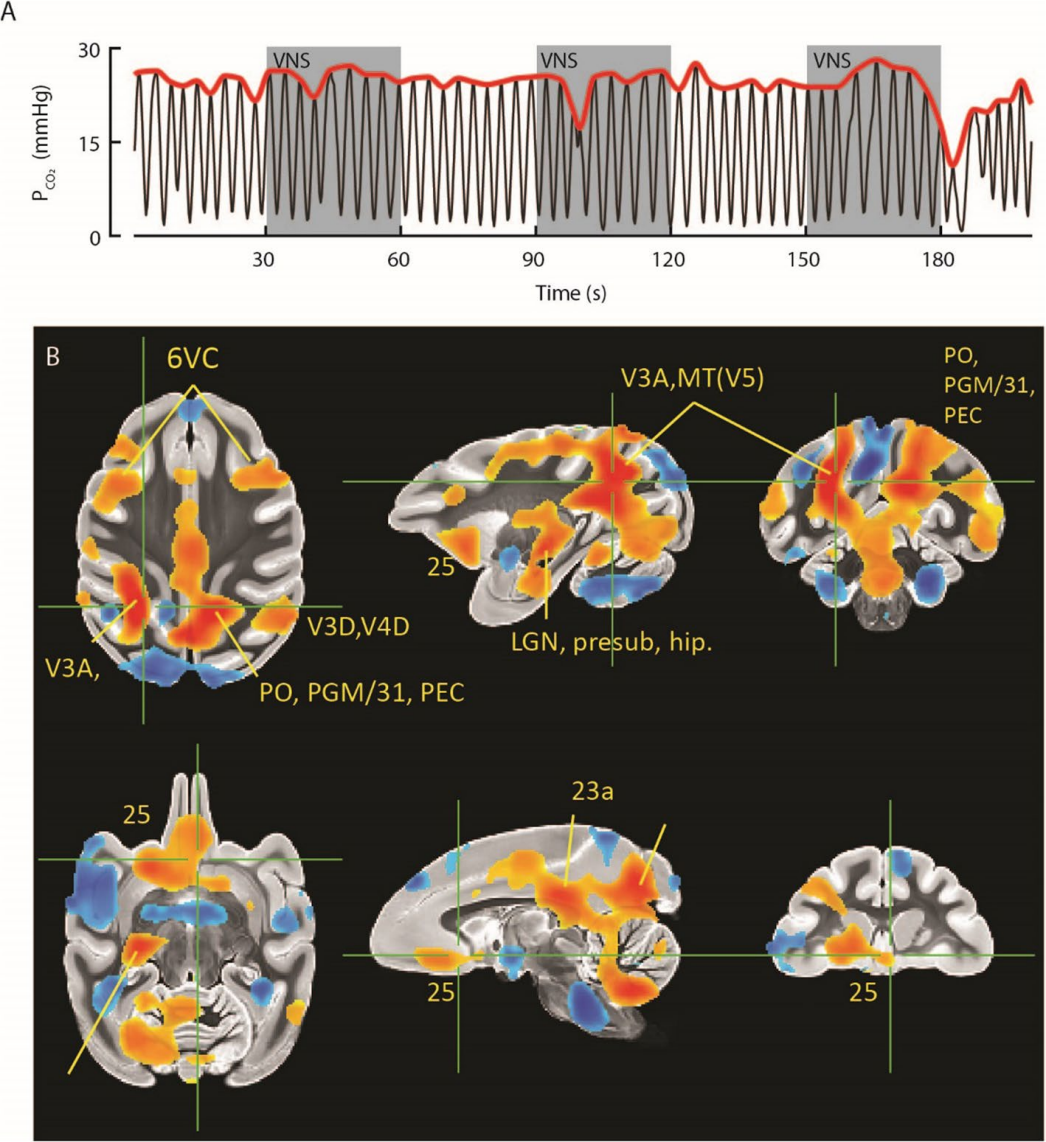
Neural correlates of VNS via functional MRI. **A** VNS applied to an awake NHP (*n* = 1) in the MRI scanner resulted in minimal interruptions of respiration consistent with the start of stimulation (gray bands). **B** BOLD fMRI measured during VNS revealed statistically significant areas of activation and deactivation in both cortical and subcortical structures. See results for a full description of activated regions

For fMRI the animal was trained to sit quietly in the bore of the scanner inside a custom-built MRI compatible primate chair for the duration of the imaging session, which lasted approximately 3 h; please see Birn et al. (2019) for a complete description of the training procedures. VNS was delivered on a 30 s on, 30 s off schedule. Consistent with our desire to avoid large, stimulation evoked changes in respiratory function during BOLD imaging, VNS at 700µA produced minimal interruption in the breathing rhythm. The breathing rhythm is illustrated by the black function in Fig. 5A, which plots PCO2 (mmHg) as a function time. The red function on the same panel represents the envelop computed as described in the methods section. The epochs of VNS presentation are denoted by the grey background blocks.

Increases in the BOLD signal that were correlated with VNS administration were documented throughout the brain. In particular, we observed increases in the precuneus area (specifically areas PGM/31, PEC, and parieto-occipital PO), occipital areas V3D and V4D, area 23a, a frontal cluster encompassing parts of areas 6VC and 6DC, an inferior frontal region encompassing area 25, a cluster in the lateral geniculate nucleus (LGN), presubiculum, and hippocampus, medial superior temporal region (MST), and middle temporal area /a visual area 5 (MT(V5)) (Fig. 5B). Decreases in the BOLD signal that were correlated with VNS administration were observed in the insular cortex. Importantly, a separate analysis showed that the end-tidal CO2 variations over time were not significantly correlated with the VNS administration timing (*p* > 0.8). Furthermore, the activation maps were similar when including either the global signal, the end-tidal CO2, or both, as additional nuisance regressors.

**Fig. 6:**
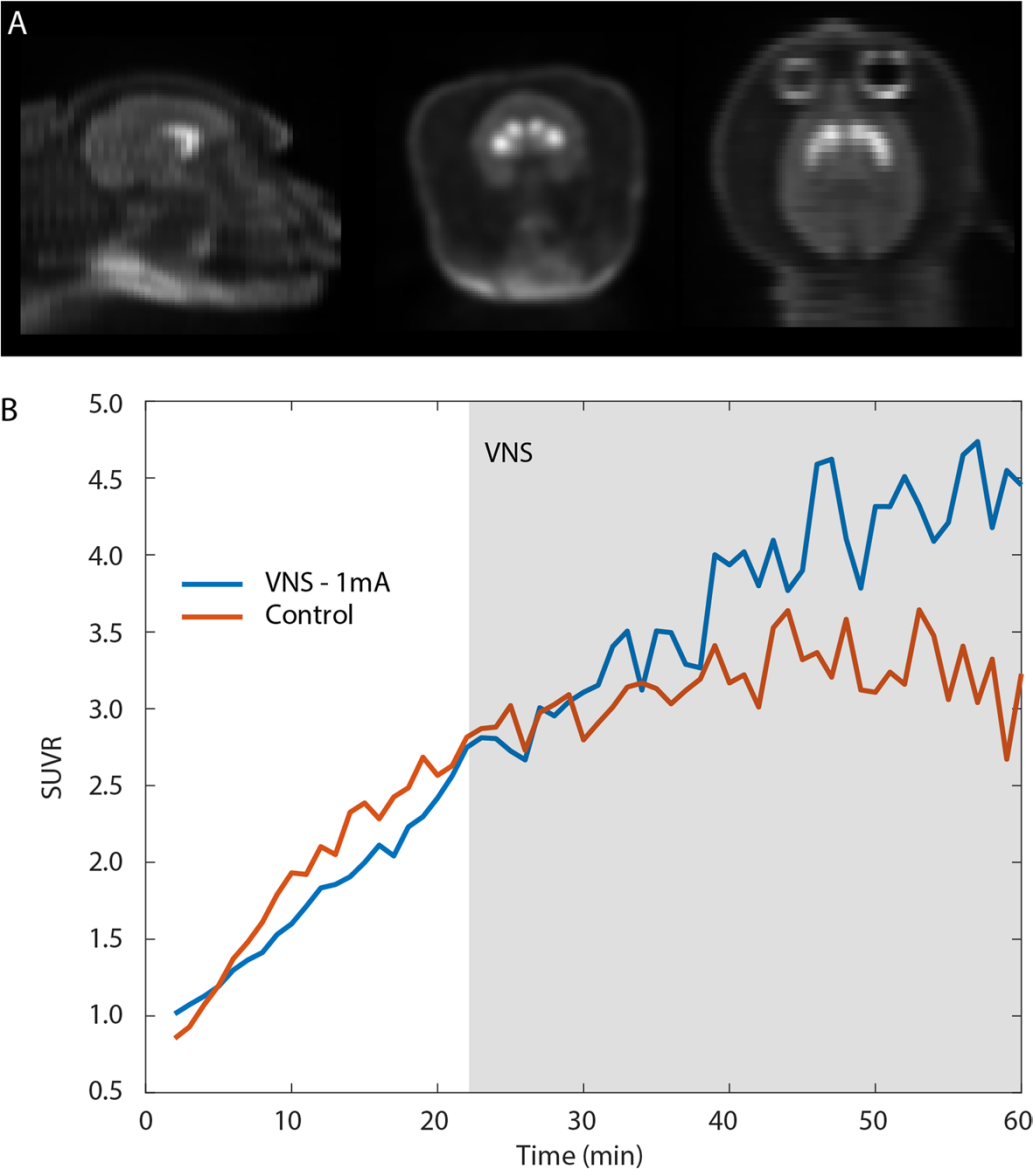
Assessment of neurochemical activity via PET. **A** Sagital, coronal and axial (left to right) PET images illustrating [^11^C]Raclopride, a selective antagonist on D2 dopamine receptors, uptake in the brain of one NHP. Consistent with its higer densisty of dopaminergic terminals, the striatum appears brighter than other regions demonstrating higher concentrations of the PET tracer. **B** Time activity curves for [^11^C]Raclopride within the striatum for VNS (blue) and control (orange) conditions. VNS increased binding of the tracer indicating a decrease of endogenous dopamine compared to control

For PET, we used [11C]Raclopride, a selective antagonist of dopamine D2/D3 receptors (Farde et al. 1995; Alakurtti et al., 2011; Svensson et al. 2019) to indirectly measure the effect of VNS on dopamine release. Figure 6A shows, from left to right, sagittal, coronal, and axial PET images of the monkey studied illustrating [11C]Raclopride uptake in the striatum, the area of the brain where highest values were observed. Figure 6B shows SUVR computed by normalizing to the cerebellum and plotted as time activity curves for a control and a VNS session (1 mA, 30 s on, 30 s off). VNS increased [11C]Raclopride binding to D2/D3 receptors (Fig. 6B, blue trace between 40–60 min), indicating a decrease of endogenous dopamine compared to the control scan (Fig. 6B, red trace). A comprehensive study, including tracers for cholinergic receptors, is currently being conducted in awake animals using the same techniques.

## Discussion

In addition to its broad use for treating intractable epilepsy (Ben-Menachem 2002; Landy et al. 1993; Handforth et al. 1998; Penry and Dean 1990) and depression (O’Reardon et al. 2006; Marangell et al. 2002; Rush et al. 2005; Sackeim et al. 2001), VNS is one of the most extensively studied neuromodulation therapies due to its potentially widespread effectiveness in treating disorders throughout the body. Specifically, it is thought to hold great potential for treating disorders of the CNS such as Parkinson’s Disease (Farrand et al. 2017), and for promoting recovery after injury (Pruitt et al. 2016; Khodaparast et al., 2013; Ganzer et al. 2018), but progress has been limited by a lack of understanding of the neurobiological mechanisms underlying its effects. More importantly, preclinical large animal studies have been performed primarily in canine and swine models (Settell et al. 2020; Blanz et al. 2023; Nicolai et al. 2020; Yoo et al. 2013; Yoo et al. 2016) which prevent the comprehensive study of VNS effects on higher order behavior and cognition because they cannot perform complex tasks. To that end, we adapted an approach to implant clinical electrodes on the left vagus nerve of Rhesus monkeys and verify appropriate target engagement in both awake and anesthetized experiments. This model, while not as similar to humans in the anatomy or the vagus nerve compared to swine (Settell et al. 2020), is able to perform cognitive tasks and is the closest animal model available to humans in structure and function of the brain (Wise 2008). We demonstrated long term viability (> 120 days, see Fig. 2D) of the implant, as well as its use in behavioral and imaging experiments examining the physiological and neurobiological basis of VNS. These efforts clearly show that a large animal model of VNS based on the Rhesus monkey is viable and provides a foundation for future studies examining the effects of VNS on autonomic, motor, and cognitive function.

Two major factors contributed to the data collected: 1) the animal preparation and the combination of existing techniques used to instrument the subjects for the delivery of VNS, and 2) the training for the study of the effects of VNS under various experimental contexts. The Rhesus monkey was chosen because its behavioral capabilities and central nervous system are most analogous to that of humans (Wise 2008) making it the animal model of choice for studies of the mechanisms underlying higher order function. It must be noted, however, that the number and organization of fascicles in the swine VN, not the Rhesus monkey, most closely resembles the properties of human VNS, making the swine a more logical choice for studies aimed at optimizing electrode design, stimulation parameters and characterizing off target effects/dose response properties to be used in humans (Settell et al. 2020; Nicolai et al. 2020; Blanz et al. 2023). The swine, however, lacks the behavioral repertoire of the Rhesus and cannot be easily imaged under awake conditions to study the effects of VNS on cognitive function. In contrast, monkeys can be trained to sit quietly in the bore of the scanner for long periods of time (Birn et al. 2019) and their cognitive performance can be readily measured using tests that are nearly identical to those employed in human studies. In addition, the neck/head anatomy of the Rhesus monkey is very similar to that of humans, which allowed us to apply Santos’ (2004) method with the modifications listed above for implanting cuff electrodes to deliver cervical VNS to humans.

### Implantation methodology and anatomical considerations

The electrode and technique for implantation and anchoring used in this work were adapted from methodology used in human patients suffering from epilepsy and depression (see Santos 2004 for a full description of the human implant procedure) and is similar to previous work in non-human primates (Rembado et al. 2021). Clinical helical cuff electrodes (LivaNova PerenniaFLEX) with a 2 mm inner diameter were well suited to the different sized rhesus monkeys used in this study. We saw no evidence of lead migration leading us to conclude that our anchoring/strain relief positioning was successful. Functionally, the chronic implants were effective as impedances remained stable over long periods of time (Fig. 4D).

The need to deliver precisely timed VNS within behavioral epochs to carry out cognitive testing required us to modify the electrodes by replacing the standard connector with one suitable for the MRI environment that could be connected to the laboratory stimulation hardware. This methodology proved successful as the electrodes remained viable over several months as demonstrated by stable impedance measurements (Fig. 4D). Only one subject experienced complications due to the implant. Specifically, the animal developed a mild superficial infection between the implant site and the connector as fluid entered the outer casing of the electrode leads that were routed to the head cap. Inspection of the area lead us to conclude that the problem resulted most likely from failure in the sealant applied to the interface area between the electrode leads and the new connector. The problem was resolved by removing the electrode, the leads, and the connector, and replacing them with a new implant after the animal had recovered. No complications were encountered with the second implant.

We found variability in the structure and anatomical organization of the rhesus VN (see Table 1), which complicates the search for standard parameters for the delivery of VNS across subjects. One half (3/6) of the VNs we examined histologically contained a single large fascicle whereas the others had a large fascicle with 1 or 2 smaller fascicles separated by epineurium. The identity of accessory fascicles is unknown as their source/target was not identified and detailed immunohistochemistry was not performed. In addition, in approximately two-thirds of the NHPs used in this study, the cervical sympathetic trunk was found lying alongside the VN and could be easily isolated prior to cuff implant. However, in the remaining, the sympathetic trunk was merged with the VN requiring delicate dissection to open the adventitia/epineurium to isolate them. Thus, it is possible that the smaller fascicles observed histologically were sympathetic in origin as the sympathetic truck follows the course of the VN through the neck and often hitchhikes or sends cross connections to the VN (Seki et al. 2013, 2014). Thus, it is unlikely that any strategy will eliminate all fibers of sympathetic origin from stimulation. Moreover, data from one of our early acute experiments support this interpretation: after initially placing the electrode around both the VN and the putative sympathetic branch, stimulation of the electrode resulted in tachycardia, which turned into bradycardia after the sympathetic trunk was excluded from the cuff. We cannot, however, discount the possibility that some accessory fascicles may be efferent in origin and branching off the VN to form the laryngeal nerve that innervates musculature in the larynx (Settell et al. 2020; Upadhye et al. 2022) as we did not measure EMG in neck/laryngeal musculature. More detailed descriptions of fascicular organization including identification of fiber type via functional or histological measures, as well as, fascicle origin, merging and termination should be topics of future research. Such studies have been valuable in optimizing effect/side effect considerations in swine models (Settell et al. 2020; Nicolai et al. 2020; Blanz et al. 2023) and would be beneficial for the NHP as well.

### Characterization of VNS target engagement

Investigations of VNS target engagement were conducted in both anesthetized monkeys during intraoperative sessions and in awake monkeys in the laboratory. The primary outcome measures of target engagement in these experiments were changes in cardiovascular or respiratory function as bradycardia/tachycardia and depressed respiratory function are often reported as a consequence of VNS (Hatridge et al. 1989; Sturdy et al. 2017; Saku et al. 2014). During intraoperative sessions, VNS was accompanied by decreases in respiration rate that began approximately 15 s following the start of the 30 s stimulation epoch and recovered the baseline value approximately 20 s after stimulation ceased. The current amplitude to evoke respiration drops greater than 20% with respect to baseline varied across monkeys ranging from 0.8 – 1.2 mA. Below those levels, changes in respiration were variable and did not always cross the 20% threshold. We found no significant change (i.e., less than 20% deviation on average) in heart rate or SPO_2_ as a result of VNS at simulation amplitudes that caused large drops in respiration rate (see above for description of anatomical variation and its effects. This is likely due to two factors: the moderate stimulation amplitudes applied during VNS and the cuff electrode being placed on the left VN, as our results mirror those in humans implanted for left VNS (Krahl 2012; De Ferrari et al. 2017). Invasive measurements of blood pressure were variable and prone to error due to issues with the placement and function of the catheter; thus, we did not analyze these data. Our goals in these intraoperative experiments were twofold. First, early terminal experiments were used primarily to refine surgical methodology and verify target engagement to prepare for later chronic studies. Second, later procedures were in monkeys who received chronic implants and we sought to minimize the time spent under anesthesia.

Similar to the intraoperative experiments, VNS in awake monkeys with chronic implants was accompanied by significant changes in respiratory but not cardiovascular function. Effects of stimulation were variable. Short 10 s bouts of VNS were accompanied by transient depressions in respiration rate and the depth of respiration as measured by the envelope of changes in PCO_2_. Longer periods of stimulation (i.e., 30 s) often resulted in transient depressions in PCO_2_ early in the epoch that gave way to more normal function as stimulation persisted, likely due to the NHPs actively counteracting the reflex-related effects of stimulation and breathing voluntarily (Fig. 4A). Thus, we computed dose response curves for VNS using shorter stimulation epochs where the effects were more robust and consistent. There was a monotonic relationship between current amplitude and respiration rate/PCO_2_, with increases in current amplitude resulting in larger drops in these parameters. Thresholds to evoke a significant drop were consistent across NHPs. Unfortunately, we did not explore a large enough parameter space to quantify the region of the curve where saturation of the effect occurred. Interestingly, we found these effects to be more consistent for measures of PCO_2_ compared to respiration rate (Fig. S1). This was likely due to long integration times in the computation of respiration rate that didn’t adequately capture the dynamics of this experimental paradigm. A significant limitation of these data is related to the absence of measurement for side effects of stimulation. Anecdotally, higher stimulation amplitudes, above 1.2 mA, seemed to elicit some side effect as there was evidence of somatosensory effects of stimulation (i.e., NHPs touching or brushing the skin near the implant location). Future work should characterize the current level required to evoke measurable electromyograms in neck muscles.

In interpreting the physiological response to the stimulation applied through the LivaNova cuff in this study, it is important to understand recruitment order as it pertains to physiological functions mediated by the vagus. The vagus consists of different fiber types ranging from large diameter myelinated fibers to small diameter unmyelinated fibers, which is important because they have different current threshold for activation. The easiest fibers to activate (i.e. lowest current threshold) are large myelinated Aα motor nerve efferents (12–20 microns in diameter) within the vagus trunk which innervate the intrafusal motor fibers of the deep neck muscles (Dubois and Foley 1936; Foley and Dubois 1937) and Aβ fibers (6–12 microns in diameter), which are canonically somatic sensory afferents projecting from the deep neck muscles to the brainstem (Sampson and Eyzaguirre 1964) This is followed by Aγ, sensory afferents from extrafusal motor nerves ranging (3–8 microns in diameter), and Aδ, sensory afferents fibers (1–6 microns in diameter), although the overall range of Aδ fiber diameter overlaps with that of parasympathetic B fibers diameters (1–3 microns in diameter), and therefore are thought to have similar thresholds for activation. In addition to nociceptive fibers, Aδ fibers are also a class of sensory afferents leading from baroreceptors and chemoreceptors linked to VNS induced heart rate and breathing rate changes. In contrast B fibers are either 1) the vagus parasympathetic efferent innervation to the heart that have been most consistently linked to decreases in heart rate effect, or 2) pre-ganglionic sympathetic B fibers embedded or connected from the nearby sympathetic trunk that cause countervailing increased heart rate. Activation of parasympathetic B fibers have been most consistently linked to the observed bradycardic effects of VNS (Rajendran et al. 2019; Qing et al. 2018). Finally, the hardest to activate C-fibers, in addition to functions in chronic pain/ inflammation, also provide sensory afferent information from mechanoreceptors/baroreceptors/chemoreceptors associated with VNS induced changes in heart rate and/or breathing.

The amplitudes used in this study were guided by seminal work demonstrating the impact of VNS on learning conducted by Kilgard and colleagues (Engineer et al. 2011; Morrison et al. 2019; Loerwald et al. 2018; Borland et al. 2016; Kilgard et al. 2018). This work began in rodents and was successfully translated to human trials leading to a recent FDA approval. In these studies 0.8 mA was phenomenologically determined to be the optimum applied current on an inverted U curve for improving performance in task based learning paradigms. However, currents needed for activation of a specific fiber types are highly dependent on overall charge density, distance from the electrode, and non-linearities such as fascicle size/epineural thickness, etc. Therefore, it is entirely possible in the NHP with a larger diameter vagus than the rodent and a more simple fascicular organization than the human would require higher current levels to engage fiber types responsible for learning/plasticity (Settell et al. 2020; Upadhye et al. 2022). Higher levels of current were attempted in multiple animals, but was often limited by visible irritation from the awake animal. This is not surprising given that the current amplitude in human patients receiving chronic, therapeutic VNS is slowly titrated up over the course of many weeks to ‘habituate’ off-target activation of neck muscles (Fisher, et al. 2021). It is impractical to implement a similar procedure in our methodology due to the externalized electrode connections and absence of an IPG. Consequently, the currents used in this study may not have been sufficient to activate B fibers necessary to induce consistent bradycardia in the animals, but may have been at the threshold for A-delta activation thereby initiating a change in respiration via the Hering-Breuer reflex. Although possible, our data are not consistent with the breathing changes driven by activation of the neck muscles, as neck muscle activation is immediate upon stimulation. Instead, the observed breathing rate reduction often took 15 s or more into the 30 s pulse train, which is stereotypical of Hering-Breuer reflex activation (Holmes and Remmers 1989; Bucksot et al. 2020) (See Fig. 1D). Our lack of heart rate response is consistent with the recent VNS NECTAR clinical trial for heart failure, where an evoked response rate was observed in only 13/106 individuals, without intolerable neck muscle activation, at the 6 and 12 month time points post-implant (De Ferrari et al. 2017; Binks et al. 2001).

## Conclusions

The procedures developed for implanting NHPs to deliver VNS under anesthetized and behaving conditions were successful based on the durability of the implants, the effectiveness with which they evoked changes in respiratory function consistent with activation of the parasympathetic system, behavioral changes, as well as changes in brain activation measured with fMRI, and changes in the release of dopamine measured with [11C]Raclopride PET. We note that the behavioral and imaging data presented are from single subjects and only intended as proof of concept. They, however, demonstrate the potential of our newly developed methodology to implant NHPs for VNS for the study of its effects on the function of the CNS. A full description of the effects of VNS on decision making, fMRI bold activation, as well as neurotransmitter system engagement measured with PET, are underway and will be reported separately upon their completion. Finally, we anticipate that this preparation will be very useful to study the mechanisms underlying the effects of VNS for the treatment of conditions such as epilepsy and depression. Nonhuman primates, unlike rodents, are evolutionarily very close to humans, hence their great translational value. Rhesus monkeys in particular have a nervous system very similar to humans, and can be behaviorally/cognitively tested with experimental tasks very similar to those used for human studies. In addition, this NHP preparation is likely to play an important role for the study of the mechanisms underlying the potential for VNS to improve learning and memory, as well as other cognitive functions, which in humans can only be studied in cases where VNS is implanted for approved medical conditions, such as epilepsy.

## Supplementary Information

Additional file 1: Figure S1. Dose response curves demonstrating changes in respiration rate as a function of VNS current amplitude for monkey Dkand An.

## Data Availability

The datasets used and/or analyzed during the current study are available from the corresponding author on reasonable request.

## Abbreviations

CNS: Central Nervous System
fMRI: Functional Magnetic Resonance Imaging
IACUC: Institutional Animals Care and Use Committee
IPG: Implantable Pulse Generator
NHP: Non-Human Primate
PET: Positron Emission Tomography
SUVR: Relative standardized uptake values
VN: Vagus Nerve
VNS: Vagus Nerve Stimulation
