# Supplementary material for: Vagus nerve stimulation in the non-human primate: implantation methodology, characterization of nerve anatomy, target engagement and experimental applications
Source: Bioelectron Med. 2023 Apr 28;9:9. doi: 10.1186/s42234-023-00111-8 (PMC10148417; doi:10.1186/s42234-023-00111-8)
Supplement: Supplementary file 1 — Additional file 1: Figure S1. Dose response curves demonstrating changes in respiration rate as a function of VNS current amplitude for monkey Dkand An. [file 42234_2023_111_MOESM1_ESM.docx]

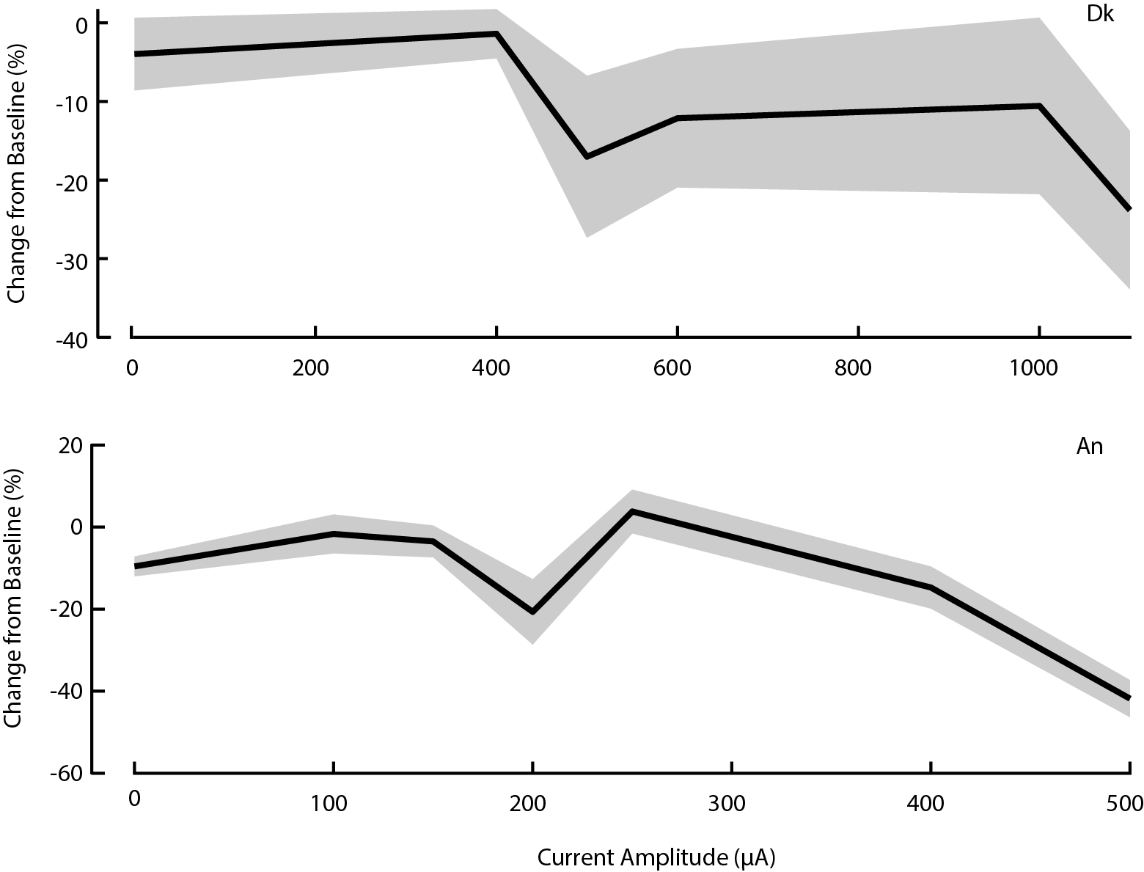


**Figure S1.** Dose response curves demonstrating changes in respiration rate as a function of VNS current amplitude for monkey Dk (top) and An (bottom).
